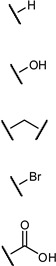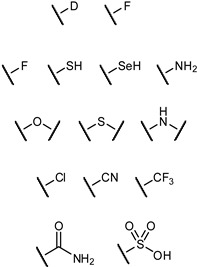# Strategies for the Development of Glycomimetic Drug Candidates

**DOI:** 10.3390/ph12020055

**Published:** 2019-04-11

**Authors:** Rachel Hevey

**Affiliations:** Molecular Pharmacy, Dept. Pharmaceutical Sciences, University of Basel, Klingelbergstr. 50, 4056 Basel, Switzerland; rachel.hevey@unibas.ch

**Keywords:** carbohydrate, glycomimetic, drug development, lectin, lead optimization, binding affinity

## Abstract

Carbohydrates are a structurally-diverse group of natural products which play an important role in numerous biological processes, including immune regulation, infection, and cancer metastasis. Many diseases have been correlated with changes in the composition of cell-surface glycans, highlighting their potential as a therapeutic target. Unfortunately, native carbohydrates suffer from inherently weak binding affinities and poor pharmacokinetic properties. To enhance their usefulness as drug candidates, ‘glycomimetics’ have been developed: more drug-like compounds which mimic the structure and function of native carbohydrates. Approaches to improve binding affinities (e.g., deoxygenation, pre-organization) and pharmacokinetic properties (e.g., limiting metabolic degradation, improving permeability) have been highlighted in this review, accompanied by relevant examples. By utilizing these strategies, high-affinity ligands with optimized properties can be rationally designed and used to address therapies for novel carbohydrate-binding targets.

## 1. Introduction

As one of the most abundant natural products, carbohydrates play many integral roles throughout our environment, for example as a metabolic energy source, a structural component of cell walls, and cellular recognition. They are present as various biological conjugates, including glycoproteins, proteoglycans, and glycolipids, and typically form a thick layer at the cell surface of approximately 10–100 Å which is referred to as the ‘glycocalyx’ [1,2]. This expression at the extracellular surface makes them ideally suited for interactions with neighboring cells and biomolecules.

**Figure 1:**
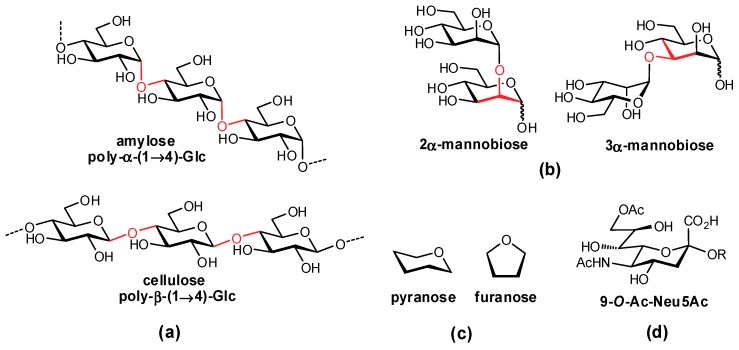
Structural variation in glycans arises from differences in: (**a**) anomeric stereochemistry; (**b**) regiochemistry of linkages; (**c**) ring size; and (**d**) further covalent modifications.

In mammals, oligosaccharides are comprised of unique combinations of a defined group of monosaccharide residues which exhibit impressive structural complexity [3]. In contrast to amino acids and nucleotides which are typically assembled in a linear fashion, carbohydrates can form both linear and branched structures with contiguous stereocenters, affording a diverse array of structures. In addition to varying stereochemistry and regiochemistry of their glycosidic linkages, the monosaccharides forming these complex structures can also vary in their ring size (e.g., furanose, pyranose) and are often further modified (e.g., acetylation, sulfation, methylation) (Figure 1). The syntheses of oligosaccharides in vivo are accomplished by carbohydrate-processing enzymes: (i) glycosylases, which hydrolyze terminal residues; (ii) glycosyltransferases, which add residues to an existing structure; and (iii) other glycan-processing enzymes, such as sulfotransferases, which structurally fine tune individual functional groups.

Carbohydrate structures attached to proteins are commonly classified as either *O*-linked (e.g., to serine or threonine amino acid side chains) or *N*-linked (e.g., to asparagine side chains). The biosyntheses of these two groups occur via two distinct mechanisms. *O*-Linked glycans are synthesized in a much more straightforward fashion: a monosaccharide is first transferred to the Ser/Thr sidechain and then other glycosyltransferases subsequently add to the structure, making it increasingly complex. In contrast, the formation of *N*-linked glycans involves the initial assembly of a complex oligosaccharide onto a phospholipid scaffold (dolichyl pyrophosphate), which then gets transferred within the endoplasmic reticulum to a protein asparagine residue; the glycan is then further elaborated through a combination of glycosidases which can partially deconstruct the original glycan construct, and glycosyltransferases which add additional sugar residues. Further functionalization of the glycan can then occur via the addition of acetate, sulfate, phosphate, or other groups to various positions of the oligosaccharide.

Proteins which bind to carbohydrate ligands can be broadly classified into enzymatic proteins (e.g., glycosidases, glycosyltransferases), lectins (non-enzymatic, signaling proteins), or glycosamino- glycan-binding proteins. Lectins play a prominent role in host recognition processes, and are primarily located at the cell surface but can also be found as soluble proteins. Lectins can be further classified into various sub-groups, such as the C-type lectins (e.g., selectins) which require Ca^2+^ ions for protein binding and play an integral role in intercellular adhesion and pathogen recognition, or I-type lectins (e.g., Siglecs) which are part of the immunoglobulin superfamily and are important for immune regulation. The affinities of carbohydrate-protein interactions are characteristically very weak, with dissociation constants (*K_d_*) in the range of micromolar to millimolar, which has typically been attributed to several factors. Firstly, they lack hydrophobic functional groups and are therefore unable to form hydrophobic interactions with the protein surface; hydrophobic interactions are typically a feature of high affinity interactions. Secondly, their affinity often relies on hydrogen-bonding (H-bonding) interactions with the protein surface and they have a difficult time competing with H-bonding from bulk solvent. Thirdly, since the relatively shallow, solvent-accessible protein binding site and polar surface area of the ligands both form extensive H-bonding networks with bulk solvent, this needs to be removed prior to ligand-protein association and results in large enthalpic penalties for desolvation. Finally, flexibility in their structures can result in high entropic penalties for protein binding.

### 1.1. Carbohydrates in Disease

Carbohydrates play an important role in a number of biological processes, such as cell adhesion, inflammatory migration, host-pathogen recognition, immune activation, and cancer metastasis [4,5,6]. Many diseases have been correlated with changes in the composition of cell-surface glycans, a direct result of the differential expression of glycosidases and glycosyltransferases in the diseased state. These altered protein expression levels cause a unique surface glycan composition (for example, hypersialylated or truncated structures) which potentially generates novel targets for disease therapies. Aberrant carbohydrate expression has been linked to various diseases such as cancer, infection (viral, bacterial, and parasitic), and immune dysregulation, among others. Modified glycan expression is not necessarily associated with disease, as it also occurs during different stages of tissue development, cellular differentiation, or inflammatory response.

In addition to aberrant carbohydrate expression, ‘normal’ glycans also play an important role in disease progression as they are often a target of invading pathogens and their associated toxins. For example, influenza uses haemagglutinin, one of its viral coat proteins, to bind cell-surface sialic acids on human cells, an essential process for facilitating entry of the virus into host cells [7]. Many bacterial toxins also target surface carbohydrates, such as the toxins from botulism, cholera, tetanus, and diphtheria, as well as plant toxins such as abrin and ricin [8].

### 1.2. Native Carbohydrates as Pharmaceutical Agents

Due to their extensive structural diversity, carbohydrates are excellent as recognition molecules and are a desirable target for drug development, but unfortunately suffer from a number of drawbacks when being considered as therapeutic ligands [9,10,11]. 

Since carbohydrates engage proteins through only low energy interactions (H-bonding, metal chelation, salt bridges, and weak hydrophobic interactions), the *K_d_* values of lectins are typically in the high micromolar to millimolar range except for a few examples (e.g., cholera toxin binds its GM_1_ ligand with approximately 1 μM affinity, and arabinose-binding protein binds arabinose with approximately 100 μM affinity) [12,13,14]. The weak interactions formed through protein binding are often unable to compensate for the steep enthalpic penalties required for desolvation of the polar substrate and shallow protein binding site, and therefore, lectins often also rely on multivalent interactions to improve binding affinities.

Native carbohydrate ligands have limited use as orally-administered therapies, since they are unable to passively cross the intestinal enterocyte layer. This passive permeation typically requires molecules of a low molecular mass, with limited polar surface area, and low numbers of H-bond donors and acceptors (in line with the Lipinski and Veber rules) [15,16]; the hydrophilicity of poly-hydroxylated carbohydrates (potentially with additional carboxylates, sulfates, etc.) prohibits this passive permeation.

Due to the ease at which bulk solvent can displace native ligands within the shallow binding sites, the *k*_off_ rates of lectin interactions are characteristically very high, contributing to very short residence times (often in the range of seconds) which contributes to their unsuitability as drug candidates. Once in the bloodstream, carbohydrates very quickly undergo renal excretion and clearance from the body, contributing to their extremely poor pharmacokinetic properties.

### 1.3. Development of Glycomimetic Drug Candidates

Glycomimetics are ‘drug-like’ compounds which mimic the structure and function of native carbohydrates, and have been thoroughly studied in the development of therapeutic candidates for both lectins and enzymatic carbohydrate-processing proteins [11,17]. Well-designed glycomimetics can impart enhanced affinities, increased bioavailabilities, and longer serum half-lives. Glycomimetic compounds can be designed to take advantage of additional interactions which are not present in the native counterpart, offering enhancements in both affinity and selectivity.

Several strategies have been used to overcome the poor drug-like character of carbohydrates, which are described within this review and accompanied by relevant examples. Strategies such as reducing ligand polarity, increasing affinity through the optimization of entropic and enthalpic binding components, ligand pre-organization, and improving pharmacokinetic parameters have all been examined. 

In order to rationally design glycomimetics, as much information as possible about the ligand-protein binding event should be collected [11]. To date, the most informative and utilized approaches rely on X-ray crystal structures, nuclear magnetic resonance (NMR) experimentation, and molecular modeling. Crystal structures provide information about the mode of ligand binding, and can convey which functional groups are essential for binding and which should be tolerant to modification. In cases where a crystal structure is not available, computational homology models can be developed to obtain further information. Saturation transfer difference (STD) NMR and transfer Nuclear Overhauser Effect (NOE) NMR have both been used to obtain key information on ligand binding. STD NMR provides insights into which functional groups are in direct contact with the protein, while transfer NOE NMR experiments provide important information on the precise binding conformation that the ligand adopts while bound to the protein, which can be used for ligand pre-organization strategies to reduce entropic binding penalties. By combining information from multiple approaches one can establish which functional groups are most important for target binding, thereby suggesting which groups can be further tuned and modified, for example through bioisostere replacement, derivatization, or deoxygenation. Protein crystal structures can also be used to identify amino acid residues in the vicinity of the binding site which can be targeted for forming additional interactions, such as aromatic residues or hydrophobic pockets. 

*In silico* approaches, useful for generating homology models, have also been developed to predict via molecular dynamics simulations which are the most relevant functional groups for ligand-protein binding; a recent publication by Sood et al. successfully ranked ligand functional groups as either ‘critical’, ‘enhances binding’, or ‘not important’, which could then be used to generate a pharmacophore and design appropriate glycomimetics [18]. Subsequent improvements in computational methods will prove very beneficial for aiding in glycomimetic design.

Apart from direct interactions between the ligand and protein surface, it is also important to consider the impact of structural waters on ligand binding. For highly constrained water molecules, often characterized by their presence in both liganded and unliganded crystal structures, they can typically be regarded as an extension of the binding site and therefore, interactions with these highly ordered waters can afford favorable enthalpic gains [19,20]. In contrast, H-bonding of the ligand to more mobile water molecules in the binding site can create significant entropic penalties by restricting the movement of the water molecule in the bound state. Several computational methods are in development to help with more accurately predicting structural water molecules and the strength of their interactions. Highly conserved water molecules have been important for considerations in developing glycomimetics against several carbohydrate-binding proteins, for example FimH and l-arabinose binding protein [19,21].

**Figure 2:**
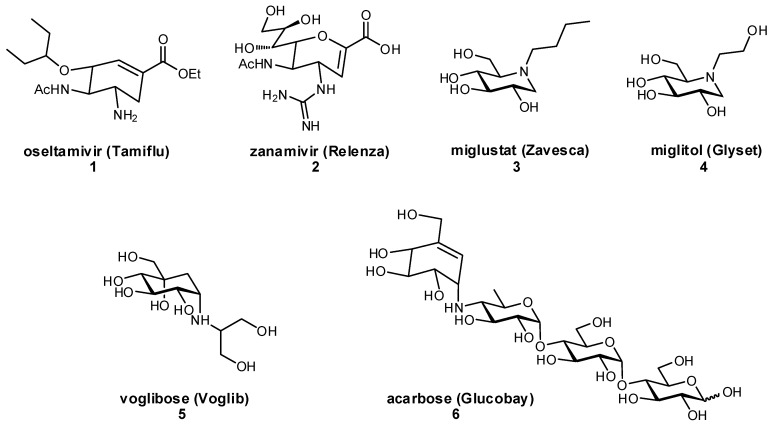
Examples of glycomimetic inhibitors that have successfully reached the market.

The development of glycomimetics has already proven successful in several cases, with multiple candidates reaching the clinic. These successes mostly target the enzymatic carbohydrate-processing proteins and have typically been based on transition state mimetics. Arguably the most widely known example, oseltamivir (Tamiflu^®^) is a glycomimetic inhibitor of influenza neuraminidase and is used in the treatment of influenza infection (Figure 2) [22,23]. Zanamivir (Relenza^®^) is another established neuraminidase inhibitor for influenza treatment [24]; both oseltamivir and zanamivir inhibit the cleavage of terminal sialic acid (Neu5Ac) residues on host cells, which is an essential process for viral propagation and thereby limits disease progression. Miglustat is an inhibitor of glucosylceramide synthase which has been used in the treatment of type I Gaucher disease to prevent the harmful accumulation of glucosylceramide [25]. Another successful family of therapeutic glycomimetics is the α-glucosidase inhibitors (miglitol, voglibose, acarbose) which have been used in the treatment of diabetes and lysosomal storage disorders [26,27,28]. These glycomimetics are again transition state mimics, and as the target protein α-glucosidase is present in the brush border of the small intestine, passive permeation of the drug through the gastrointestinal membrane is not required for drug activity. As can be seen for miglustat, miglitol, and other transition state glycomimetics which directly inhibit enzymatic processes, ionizable groups are an important chemical feature for mimicking the charged oxocarbenium transition state.

For diseases with an unmet clinical option, carbohydrates provide a promising target. In a time of increasing concern over antibiotic resistance, the emergence of anti-adhesive therapies offers a promising alternative [29,30]. Carbohydrates play an imperative role in the adherence of many pathogens to host cells, contributing to both infectivity and pathogen-avoidance of host clearance. Glycomimetic inhibitors, intelligently designed to have enhanced affinities over host cell-surface ligands, can be developed to prevent the adhesion of pathogens to host cells thereby facilitating clearance from the host; as adhesion does not affect overall survival of the pathogen, this approach is also at a considerably lower risk for developing resistance mechanisms. Since it is possible for bacterial and viral pathogens to express multiple types of adhesin proteins simultaneously, co-therapy including antibiotics and other anti-adhesives may ultimately be necessary for treatment success using this approach [31].

Interestingly, glycomimetics have also been used as antigens in carbohydrate-conjugate vaccines [32,33,34,35,36,37]. The non-native, glycomimetic structures have been observed to enhance immunogenicity, and if designed properly can elicit the production of antibodies that are cross-reactive with native glycans. Approaches to obtain glycomimetics with bioisosteric functional groups and glycosidic linkages have both been used in efforts toward vaccine development.

## 2. Glycomimetic Design – Strategies to Improve Binding Affinities

### 2.1. Deoxygenation

Several examples of glycomimetics have illustrated that a reduction in ligand polar surface area can enhance binding affinities by both generating new hydrophobic contacts with the protein, as well as reducing the enthalpic cost of ligand desolvation. Therefore, the removal of polar functional groups uninvolved in protein binding, most commonly the hydroxy moieties, has been well demonstrated to enhance binding affinities.

The thermodynamics of ligand-protein binding can be quantified by calculating the Gibbs free energy of an interaction, ΔG (Equation (1)), from its individual enthalpic (ΔH) and entropic (TΔS) terms, where a negative free energy is essential for productive binding events:

ΔG = ΔH − TΔS(1)

To better understand the high enthalpic cost of desolvation, one can compare the thermodynamic quantities calculated by Cabani et al. [19,38]. The enthalpic penalty of desolvating a single hydroxy group, ΔH = 35 kJ/mol, is only partially offset by the favorable entropy term that results from the release of structured water molecules into bulk solvent, ΔS = 10 kJ/mol. This results in a net free energy of +25 kJ/mol, which cannot be compensated for by the energy gain afforded by a single H-bond (approx. ΔG = −18 kJ/mol). Although vicinal hydroxy groups experience a somewhat reduced desolvation penalty in comparison to individual hydroxy moieties (approx. ΔG = 34 kJ/mol for two vicinal hydroxy groups), the high enthalpic penalty of desolvation is still unfavorable for a binding event. This suggests that a minimum of two H-bonds should form between a ligand hydroxy group and the protein in order for the free energy of binding to be considered favorable. As exemplified in the literature, the removal of hydroxy groups forming only a single H-bond with the protein binding site typically enhances binding affinity. In order to optimize affinities in the design of glycomimetic ligands, the aim should be to form a larger number of high-quality H-bonds between each of the ligand’s polar groups and the protein surface.

Although the desolvation penalty is often very high for carbohydrate-binding proteins, resulting in part from shallow and solvent-exposed binding sites, proteins with deeper binding pockets are inherently more hydrophobic, less solvated, and therefore often display enhanced affinities. In these hydrophobic binding cavities, the H-bonds between ligand and protein are considerably stronger (approx. 10-fold), experience less competition from bulk solvent, and also have improved residence times (reduced *k*_off_ rates) [19,39,40,41,42]. In these particular cases, where a lower desolvation penalty exists in combination with a higher enthalpic gain per H-bond, the requirement for generating such extensive H-bonding networks is reduced. 

Alternative strategies have been used to reduce ligand solvation and thereby minimize the desolvation penalty: although not exemplified with a glycomimetic, Gao et al. nicely illustrated that the addition of a hydrophobic group in a non-binding, non-relevant position of a ligand was successful in disrupting water structure around the ligand and, therefore, reduced the enthalpic cost of desolvation [43]. Even though this portion of the molecule displayed no interactions with the protein surface, it was successful in enhancing the free energy of binding.

Deoxygenation can also provide other beneficial effects. By reducing overall polarity of the molecule, this can increase the electron density on the pyranose ring and thereby enhance nucleophilicity of its remaining hydroxy groups. This enhanced nucleophilicity can strengthen interactions involving complexation of metal ions or salt bridges. Alternatively, deoxygenation of 6-OH groups removes a rotational degree of freedom yet still leaves the C-6 methyl group intact for influencing ^4^*C*_1_/^1^*C*_4_ pyranose conformational preference, which can further reduce the entropic costs associated with ligand binding.

**Figure 3:**
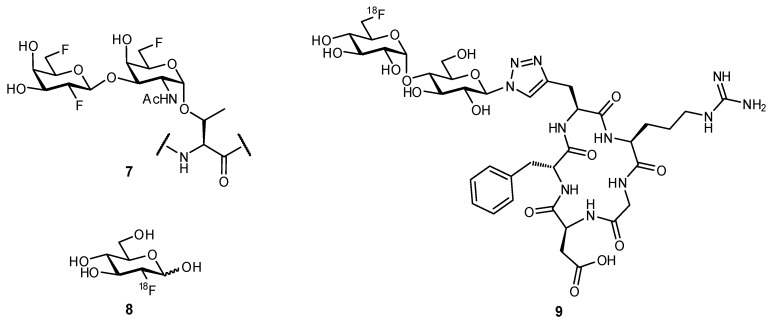
The bioisosteric replacement of hydroxy substituents with one or more fluorine atoms has proven successful in generating glycomimetics [46,48].

Replacement of a hydroxy group with a fluorine atom has been used to experimentally probe the necessity of individual hydroxy moieties for H-bonding. This same strategy of OH → F substitution can also be applied in computational modelling. The fluorine atom is useful as a bioisosteric mimic of the hydroxy group, yet is also more hydrophobic and therefore, can retain important characteristics of the hydroxy moiety yet reduce polar surface area of the ligand [44]. Several studies have revealed that upon fluorine substitution, recognition of the mimetic by its native receptor is still possible; for example, in studying the transport of d-glucose across the endothelial membrane of red blood cells, 2-deoxy-2-fluoroglucose and 3-deoxy-3-fluoroglucose afforded very similar transport rates as compared to the native ligand [45]. In another study, fluorinated mimetics of MUC1-based glycopeptides were observed to be cross-reactive with serum antibodies from mice that had been vaccinated with native antigen (compound **7**; Figure 3) [46]. This widespread recognition of fluorinated glyco-analogues has been a contributing factor to the success of ^18^F-2-deoxy-2-fluoro-d-glucose (**8**) as a radiotracer for diagnosing neoplasia through positron emission tomography (PET scans) [47,48].

### 2.2. Biomimetic Replacement of Functional Groups

Biomimetic functional groups, i.e., those with comparable electronic and steric properties, can sometimes be used to replace existing functional groups to improve properties of a drug candidate. Bioisosteric replacement is a common practice in medicinal chemistry, with many families of bioisosteres having been reported and evaluated (Table 1). Given the specific requirements for a functional group in a particular binding event (e.g., steric restrictions, H-bond donor/acceptor properties), different bioisosteres can be considered as suitable replacements in different situations.

**Figure 4:**
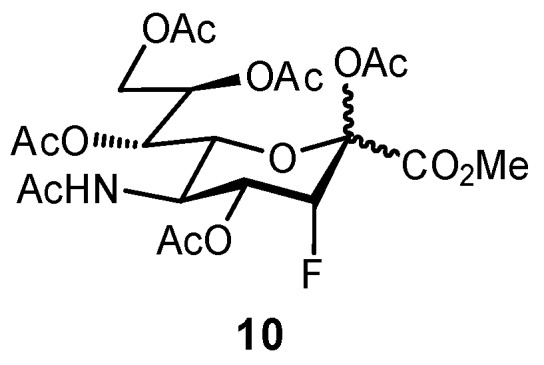
A Neu5Ac-based glycomimetic **10** was successful in preventing tumor metastasis in a mouse model [50].

Bioisosteric replacement can afford enhanced affinities in a number of ways; for example, in the previously described OH → F substitutions [45,46,49], the fluorine atom can still facilitate polar interactions with the protein surface yet reduces overall hydrophilicity of the ligand. The fluorine atom can also be used as a suitable replacement for hydrogen, owing to its small size and relative hydrophobicity; replacement of the axial C-3 proton of sialic acid (Neu5Ac), to afford the glycomimetic **10** was successful in generating an inhibitor of sialyltransferase (Figure 4) [50,51]. Substitution with the fluorine atom afforded a ligand which was sterically compatible with the binding site, yet the unique electronic properties of fluorine generated a much more electrophilic anomeric carbon (C-2), improving antagonist ability. To overcome the negligible oral availability associated with such a polar substrate, the drug candidate was peracetylated; treatment in mice successfully impaired the progression of murine melanoma by inhibiting the attachment of metastatic cancer cells to the extracellular matrix and was also observed to slow down tumor growth in vivo.

In alternative biomimetic approaches, hydroxy groups binding to an active-site metal ion can be replaced with improved metal ligands, assuming that this modification is well tolerated by the binding site. Non-covalent interactions between sulfur and π-systems are typically stronger than those with oxygen atoms, suggesting a suitable route for further enhancing binding enthalpies. Aside from enhancing the enthalpic and entropic contributions of binding, bioisosteric replacement can also be useful for the removal of groups prone to metabolic degradation, or those that facilitate rapid excretion; these effects on the pharmacokinetic properties of a drug candidate will be discussed in more detail later.

### 2.3. Targeting Neighboring Regions of the Binding Site

For lectins with a well-structured binding pocket (which facilitates reduced entropic penalties upon generating additional interactions), it can be beneficial to look for new, enthalpically-favorable binding opportunities. The most promising approaches have targeted nearby aromatic or aliphatic residues and hydrophobic pockets, since ligand modification with hydrophobic groups has the added advantage of reducing the overall polar surface area of the ligand. Although, in general, hydrophobicity is preferred, additional interactions with neighboring ionic groups can also be realized, either through salt bridges or cation-π interactions. The overall approach for developing high-affinity glycomimetics is to optimize the individual entropic and enthalpic binding contributions; the majority of efforts in developing carbohydrate derivatives have focused on targeting surrounding protein sites that can both positively enhance binding contributions and also improve ligand selectivity against a particular target, with some examples highlighted below.

**Figure 5:**
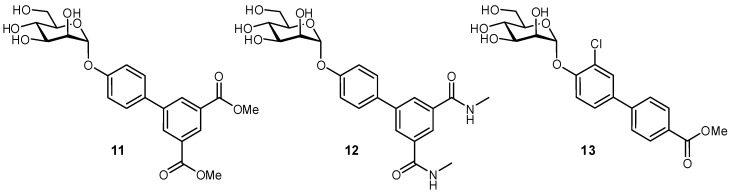
Biaryl mannosides have been successfully developed as nanomolar antagonists of the bacterial protein FimH [56,60].

A large body of work has been focused on developing FimH antagonists, as an anti-adhesive approach to treating urinary tract infections (UTIs). UTIs are one of the most common causes of infection in developed countries, typically caused by uropathogenic *Escherichia coli* bacteria [52,53]. Antibiotic resistance has been of increasing concern for treating these infections, and therefore, the possibility of anti-adhesive treatment offers a promising alternative. Type 1-fimbriae on *E. coli* facilitate bacterial adherence to the bladder epithelium and enable the pathogen to avoid clearance during micturition; the FimH protein is located at the tip of the fimbriae, and binds to the highly mannosylated glycoprotein uroplakin 1a present at the epithelial surface [54,55]. Examination of the FimH crystal structure was very beneficial for glycomimetic development, as it provided pertinent information on the ligand binding mode and also suggested further modifications to improve ligand affinity [55]. It was observed that the 2-, 3-, 4-, and 6-OH groups of the d-mannose residue form an important H-bond network in the buried ligand cavity with amino acid side chains Asp54, Gln133, Asn135, and Asp140, and backbone atoms from Phe1 and Asp47. Not unexpectedly, attempts to modify these positions have generally proven unsuccessful. Alternatively, the region surrounding the binding site entrance contains two tyrosine residues and one isoleucine residue (Tyr48, Ile52, and Tyr137), often referred to as the ‘tyrosine gate’, which can form hydrophobic contacts with glycomimetics and have been a major target for improving the affinities of FimH antagonists. First developed were aryl and *n*-alkyl mannosides, which displayed increased affinities due to interactions with a hydrophobic rim surrounding the deep binding pocket and the aforementioned tyrosine gate. The groups of Janetka and Hultgren improved affinities by using 4′-biaryl mannosides with a meta substituent that could act as an H-bond acceptor (**11** and **12**), in which the aromatic extension formed an optimal π-π interaction with Tyr48 and a new H-bonding electrostatic interaction with Arg98/Glu50, resulting in nanomolar binding affinities (Figure 5) [56]. Contributions from many groups in the development of α-mannosides and oligomannosides have improved FimH antagonists even further [57,58,59,60,61,62].

**Figure 6:**
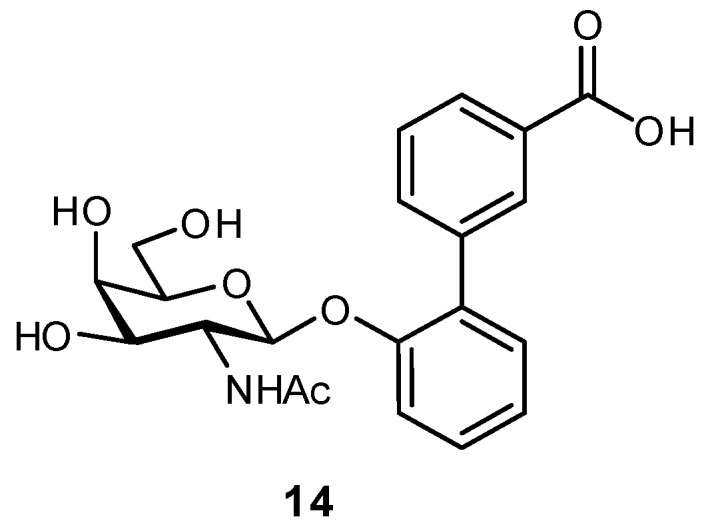
Biaryl glycosides have also been developed as antagonists of the adhesin FmlH; co-treatment with FmlH and FimH antagonists in a mouse model of urinary tract infection significantly facilitated bacterial clearance [31].

The targeting of neighboring residues has also been used in the development of antagonists for FimH-like adhesin (FmlH) [31]. FmlH is a pilus adhesin which binds galactosides and *N*-acetyl-galactosaminosides presented on bladder and kidney tissue, facilitating the adhesion of *E. coli* to these surfaces. In efforts to inhibit this interaction, aryl galactosides and *N*-acetyl-galactosaminosides were designed which facilitated several key protein interactions: a π-π interaction with Tyr46, a salt bridge between the carboxylate and Arg142, and a H_2_O-mediated H-bond between the *N*-acetyl group and Lys132. The best inhibitor (**14**) displayed a *K*_i_ of approximately 90 nM and upon administration in a mouse model was able to reduce the bacterial load in both the kidney and bladder (Figure 6). Co-treatment with a FimH antagonist further improved bacterial elimination.

**Figure 7:**
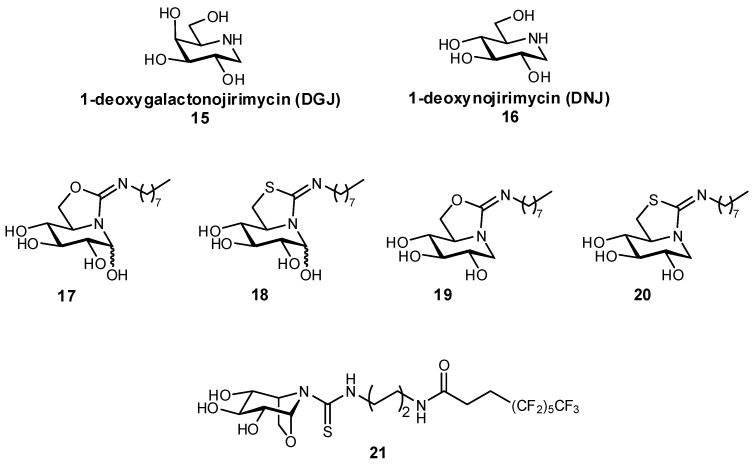
Iminosugars have been used as pharmacological chaperones, in efforts to treat lysosomal storage diseases [63,66].

In addition to the aforementioned glucosylceramide synthase inhibitor miglustat, iminosugars have also been developed as protein chaperones with picomolar affinities for the treatment of Gaucher disease, the most prevalent lysosomal storage disease (LSD) [63]. LSDs ultimately result from a glycosidase deficiency, as glycosidases are important for the break-down of lysosomal glycosphingolipids. In LSDs such as Gaucher disease and Fabry disease, genetic mutations result in the misfolding of proteins, which are then targeted for degradation in the endoplasmic reticulum instead of being trafficked to the lysosome, resulting in significantly reduced lysosomal concentrations of protein. In pharmacological chaperone therapy, sub-inhibitory concentrations of a protein ligand can be used to stabilize the protein conformation, enabling successful trafficking of the protein to the lysosome; if designed appropriately, upon reaching the lysosome the protein should bind with higher affinity to its native ligand (also present in larger excess), thereby still retaining its native activity. In order to be effective as molecular chaperones, these glycomimetics should both be selective for their target, as well as reach the endoplasmic reticulum. This approach has previously been demonstrated for the glycomimetic 1-deoxygalactonojirimycin (Migalastat^®^; Figure 7), an inhibitor of α-galactosidase in vitro, which has been successfully used as a pharmacological chaperone in the treatment of Fabry disease [64,65]. Alternatively, glycomimetic inhibitors based on 1-deoxynojirimycin (DNJ) have been developed by Mena-Barragán et al. and García-Moreno et al. in the development of a therapy against Gaucher disease (Figure 7), which results from a β-glucocerebrosidase deficiency [63,66]. Modification of DNJ to form sp^2^-iminosugars significantly enhanced targeting to the endoplasmic reticulum, and even more fortunately the ligands were found to have enhanced binding at neutral pH over acidic pH, which suggests that their affinity will decrease after entering the lysosome which should aid in protein dissociation and reduce competition with its native substrates. The iminosugars were found to successfully act as molecular chaperones for proteins expressed in mutated G188S/G183W fibroblasts (a disease-associated genetic mutation); for example, structure **20** afforded a more than 70% increase in protein activity at only 20 pM concentration, and a 300% improvement at a 2 nM concentration.

**Figure 8:**
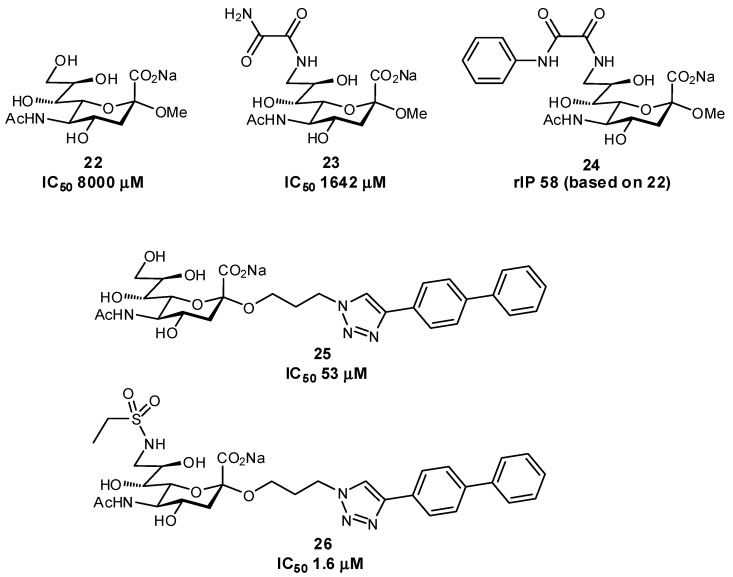
Inhibitors of Siglec-7 which target additional protein interactions through modification at C-2 and C-9 positions (rIP = relative inhibitory potency) [67,68].

**Figure 9:**
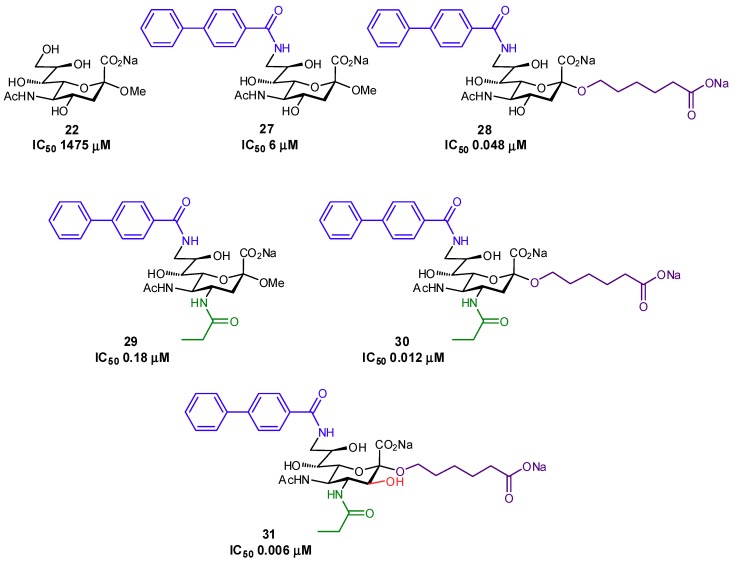
High-affinity inhibitors of Siglec-2 generated through C-2, C-4, and C-9 modification of sialic acid [70].

Several other successful examples of ligand modification have been used to enhance the affinities of glycomimetics for their protein target. For example, Siglec-7 inhibitors have been synthesized which contain C-9 aromatic modifications (also targeting a ‘hydrophobic gate’ observed in the crystal structure) and/or triazole-containing hydrophobic groups at C-2 of Neu5Ac, in an effort to develop inhibitors which could prevent immune evasion by cancer cells (Figure 8) [67,68]. Similar in structure, Siglec-2 (also known as CD22) Neu5Ac glycomimetics containing a C-9 *N*-aromatic moiety, C-4 *N*-acyl derivative, and C-2 *n*-alkyl group have been used as inhibitors and towards drug conjugates to specifically target uptake into specific subsets of immune cells via Siglec-2-binding clathrin-mediated endocytosis (Figure 9) [69,70,71]. *Pseudomonas aeruginosa* lectin B (LecB) inhibitors have been developed in an effort to tackle biofilm formation: low molecular weight, nanomolar affinity ligands with good kinetic and thermodynamic properties were developed by targeting a hydrophobic patch on the protein [72]. Additionally, much work has been focused on DC-SIGN antagonists as anti-adhesives, by targeting a hydrophobic groove on the protein [73].

**Figure 10:**
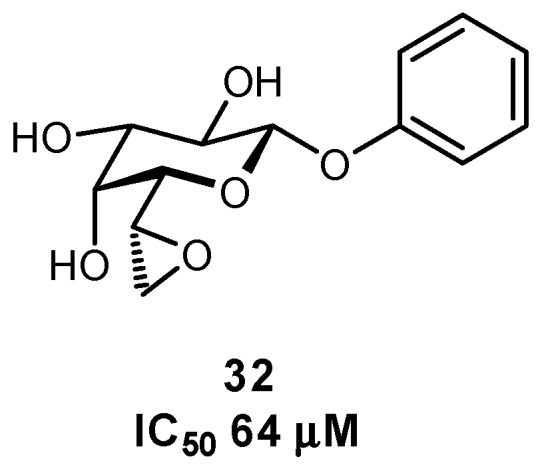
The first reported covalent lectin inhibitor, targeting *Pseudomonas aeruginosa* LecA [74].

A novel approach which also targets neighboring residues of a lectin binding site has been the development of a covalent lectin inhibitor against LecA of *Pseudomonas aeruginosa* [74]. Both LecA and LecB virulence factors have been associated with biofilm formation; although high affinity inhibitors against LecB have been developed, LecA has proven a more challenging target. In order to overcome the large *k*_off_ associated with LecA-ligand interactions, thereby enhancing affinity, a covalent inhibitor was developed which targets a nearby cysteine (Cys62) residue (Figure 10). This use of a covalent inhibitor attempts to circumvent the inherently weak affinities which arise from the short lifetimes of lectin-ligand complexes by permanently appending the ligand to the protein.

### 2.4. Conformational Pre-organization

Improvements in binding affinity through pre-organization have been successful in a number of glycomimetics [75,76]. Pre-organization reduces the entropic penalties associated with binding and additionally tends to reduce polar surface area since internal polar groups interact amongst each other, effectively shielding them from bulk solvent. Molecules have inherent entropy when free in solution, related to both translation and rotation (including internal rotation at single bonds). Entropic costs are associated with the binding of ligands, since a restriction of motion occurs through both a loss of rotational and translational entropy (for both ligand and protein); the greater the rigidity of the formed complex, the higher the entropic penalty of binding [77,78].

As mentioned previously, productive binding can only occur with a negative free energy; this requires that the unfavorable entropic costs from restriction of the binding site be offset by favorable intermolecular interactions of ligand binding, considering both enthalpic contributions (e.g., H-bonding, van der Waals, etc.) and entropic contributions (e.g., release of water molecules from the binding site). Flexible receptors which require an ‘induced fit’ binding mode suffer from even greater entropic binding penalties, since the protein loses much of its conformational flexibility, therefore requiring even greater enthalpic compensatory interactions to enable productive binding events.

Pre-organization has been shown to play an important role in the development of glycomimetic inhibitors. In the amino-glycosides, distortion of the pyranose ring has been used in efforts to mimic the flattened shape of the enzymatic transition state. This conformational distortion can be accomplished using a variety of approaches, such as the introduction of an sp^2^-hybridized center, modification of the ring size, or by generating bicylic or bridged systems [13]. 

The importance of conformational pre-organization has also been observed in the generation of LecB inhibitors. Glycan screening indicated that the Lewis A (Le^a^) trisaccharide, β-d-Gal-(1→3)-[α-l-Fuc-(1→4)]-d-GlcNAc, bound LecB with a *K_d_* of 220 nM [79]. Attempts to simplify the structure eliminated the d-galactose moiety entirely to afford the disaccharide α-l-Fuc-(1→4)-d-GlcNAc, but unfortunately isothermal titration calorimetry (ITC) experiments indicated a significantly reduced binding affinity resulting from an increased entropic penalty [80]. To further simplify the construct and reduce flexibility, α-l-fucosides were modified with heterocyclic aglycone substituents to afford substrates which, in some cases, could bind with affinities similar to those of Le^a^ [81].

Another successful example of pre-organization was illustrated in the development of an E-selectin antagonist. The native ligand of E-selectin, sialyl Lewis X (sLe^x^), binds with six solvent-exposed H-bonds and a salt bridge [11,82,83]. In efforts to improve the affinity of sLe^x^, a glycomimetic antagonist was developed which could be pre-organized into the binding-site conformation, minimizing the entropic penalties associated with binding. Based on the crystal structure, it was observed that the *N*-acetyl-d-galactosamine moiety does not form direct contacts with the protein, but instead only acts as a linker between the other residues; therefore, it was replaced by a non-carbohydrate moiety that linked the d-galactose and l-fucose residues in a correct spatial orientation [84]. By strategically placing substituents on the linker, the structure could be even more rigidified to further improve pre-organization and thereby also antagonist affinity [85]. With later iterations, the Neu5Ac moiety was replaced by (*S*)-cyclohexyl lactic acid which even further rigidified the glycomimetic conformation [84].

**Figure 11:**
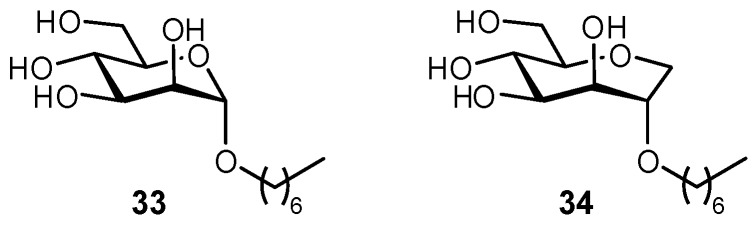
*n*-Heptyl pyranose and septanose FimH ligands have been used to study the effects of pre-organization on binding thermodynamics [86].

The importance of pre-organization has also been demonstrated in the development of FimH antagonists, upon comparing septanose versus pyranose glycomimetic scaffolds (Figure 11) [86]. In an examination of binding to the conformationally rigid FimH lectin domain, the highly flexible septanose derivative resulted in a 10-fold affinity loss. NMR, X-ray crystal structure, and molecular modeling all indicated that the related septanose and pyranose derivatives formed a superimposable network of H-bonds, yet the septanose displayed lower affinities; ITC confirmed that this loss of affinity resulted from an entropic penalty arising from flexibility of the septanose core.

### 2.5. Multivalency

Numerous glycomimetics have incorporated multivalency in order to better mimic the multivalent presentation of native ligands [81,87,88,89,90,91]. Multivalency can improve binding affinities in several ways: (i) chelation; (ii) statistical rebinding effects; or (iii) clustering of soluble binding partners [92,93]. The design of multivalent scaffolds must be carefully considered in order to incorporate proper spacing and flexibility, enabling a correct fit of the ligand into the binding site, yet concomitantly minimizing the entropic costs of binding. In general, flexible scaffolds are often more forgiving if poorly designed, but suffer from much greater entropic penalties upon binding. A recent study from the Hartmann and Lindhorst groups has also nicely demonstrated that tuning scaffold hydrophobicity can also play a significant role in the affinity of multivalent constructs [94].

**Figure 12:**
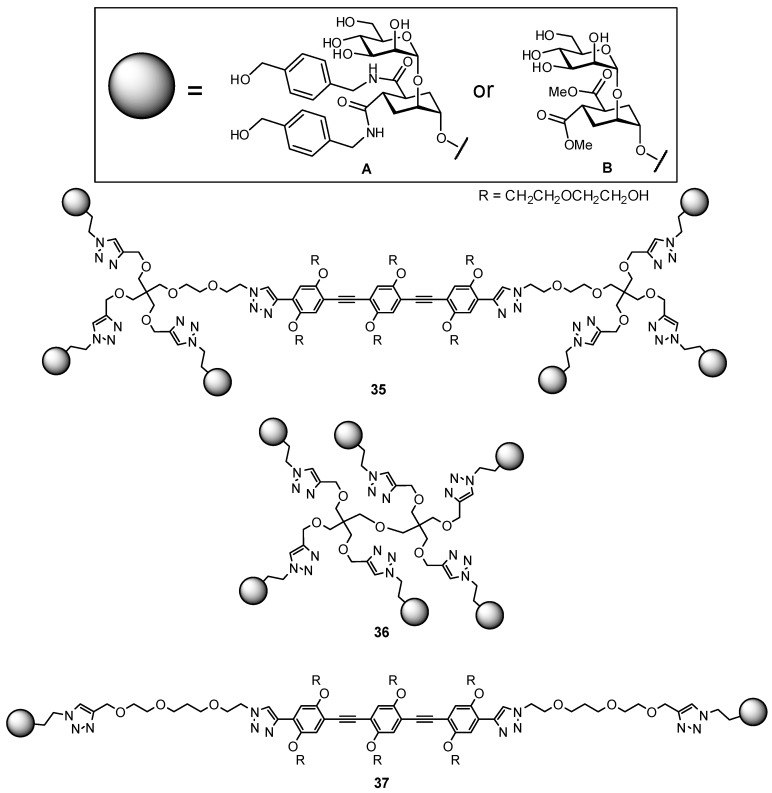
Multivalent constructs based on a DC-SIGN glycomimetic antagonist, used to probe the effects of chelation versus statistical rebinding multivalency effects [93].

In an elegant study, DC-SIGN glycomimetic antagonists were conjugated to oligovalent molecular rods and used to study multivalency effects of binding, affording nanomolar antagonists (Figure 12) [93,95]. The constructs contained a rigidified core based on phenylene-ethynylene units (previously used in the generation of *P. aeruginosa* LecA inhibitors), and were designed to be an ideal length (approx. 4 nm) for chelation to bridge carbohydrate recognition domains on neighboring DC-SIGN subunits. The length of the rigid core could be controlled, with the rigidity effectively reducing entropic binding penalties, while the ends of the rods contained trivalent constructs which had been assembled using short, more flexible linkers. The incorporated trivalent groups were intended to address favorable statistical rebinding, with the flexible linkers aimed at facilitating a better fit of ligand into the binding site (at a minor entropic cost). This intelligent design (with appropriate control compounds) was able to probe the different effects of ligand, rigid rod, and proximity effects individually.

Various other multivalent constructs have been generated in the development of carbohydrate-based pharmaceuticals, often with a lead monovalent glycomimetic being incorporated into a polyvalent construct at a later stage of project development. Multivalent constructs have targeted fucose-binding pathogenic soluble receptors, in efforts to improve the outcome of patients with cystic fibrosis, or alternatively to generate simplified mimetics of sLe^x^ that can mimic its native structure yet are easier to access synthetically [87].

## 3. Glycomimetic Design—Strategies to Improve Pharmacokinetic Properties

It has been well established that native carbohydrates display inherently poor pharmacokinetic properties [11,17]. They are sensitive to hydrolysis both in the gut and by endogenous proteins, they are not orally available, are rapidly excreted upon entering the bloodstream, and have very short receptor residence times. Many strategies have been used to improve these properties, with some overlap from the approaches used for affinity enhancement, and have been briefly outlined below.

### 3.1. Preventing Glycosidic Hydrolysis

Several approaches have been used in an effort to slow down the metabolic degradation of oligosaccharides, which are prone to hydrolysis both in the acidic environment of the gastrointestinal tract, as well as through endogenous enzymes (digestive, plasma, and cellular glycosidases). A major focus has been on generating *O*-glycoside mimetics, most commonly by replacing the bridging glycosidic oxygen atom with a more stable carbon atom, or alternatively by adding electron-withdrawing groups to the pyranose core to destabilize the oxocarbenium intermediate required for degradation. The metabolic stability of glycomimetics can be evaluated by examining the rate of degradation with serum or liver microsomes.

*C*-Glycosides can be used to improve the hydrolytic stability of oligosaccharides, but can also introduce new challenges due to an enhanced conformational flexibility, primarily resulting from a loss of the *exo*-anomeric effect [96]. Although the *exo*-anomeric effect cannot control the aglycone conformation in *C*-glycosides, in a number of examples steric bulk has been used to better restrict the aglycone unit in a gauche conformation, similar to that observed in *O*-glycosides [97,98,99]. There is indeed greater conformational flexibility observed, but it is evidently not as detrimental to binding as would be expected. Modification of the linked carbon atom with one or two fluorine atoms has also been used to enhance the electronegativity of the bridging unit and further limit its conformational flexibility, yet retain the benefit of the metabolically stable *C*-glycoside [100,101,102]. *C*-Glycosides have been used in the glycomimetic design of numerous inhibitors, including sLe^x^ [96], GM_4_ ganglioside [103], galactopyranosides [104,105], mannopyranosides [105,106,107], fucopyranosides [105,108,109], and pseudoglycopeptides [106], among others. 

**Figure 13:**
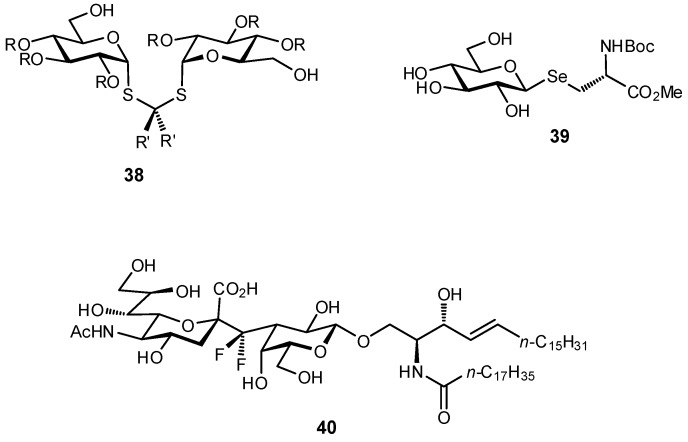
Examples of different *O*-glycoside mimetics which have been obtained synthetically, with the general aim of reducing hydrolytic degradation rates in vivo.

In addition to the *C*-glycosides, other atoms or groups have been used to substitute the glycosidic linkage, aiming for an ideal balance between hydrolytic stability and conformational pre-organization. These include various *N*-linked glycosides, as well as selenium, sulfur, and even dithioacetal analogues (Figure 13) [32,110,111,112,113,114].

**Figure 14:**
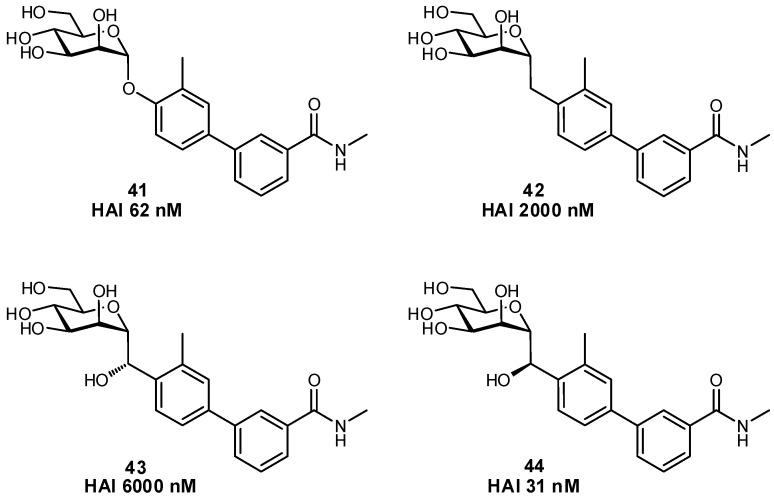
The development of *C*-glycosides for treatment of UTIs has been used to improve the metabolic stability of biaryl FimH antagonists (HAI = hemagglutination inhibition assay) [115].

**Figure 15:**
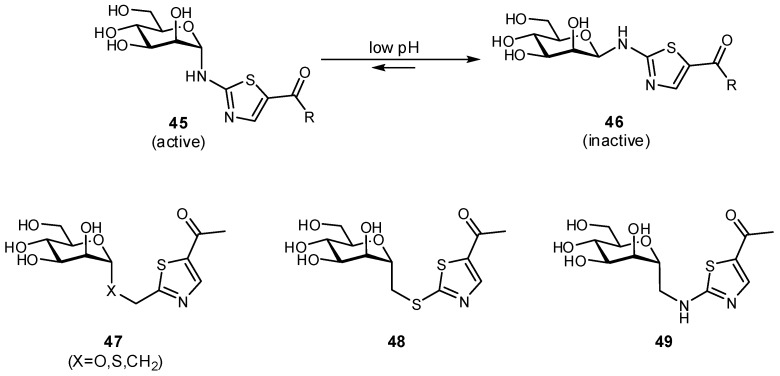
A comparison of *O*-, *N*-, *S*-, and *C*-glycosides as FimH antagonists for therapy in Crohn’s disease [116,117,119].

The biaryl mannoside antagonists of FimH for UTI therapy discussed previously were observed to suffer from very low bioavailability and rapid degradation in vivo, presumably due to metabolic instability of the *O*-glycosidic linkage in the acidic milieu of the stomach and intestinal tract, as well as to enzymatic mannosidases [115]. In order to improve pharmacokinetic parameters, *C*-glycoside derivatives were synthesized which indeed displayed enhanced stability and, in some cases, even improved inhibition (Figure 14); this improvement resulted in better efficacy in mouse models of both acute and chronic urinary tract infection. Replacement of the glycosidic linkage was also used for FimH antagonists developed as a potential therapy for patients affected with Crohn’s disease, in an effort to reduce the bacterial load of adherent-invasive *E. coli* in the ileal mucosa [116]. Thiazolylaminomannosides were synthesized with an anomeric *N*-linked aryl moiety that could form a favorable interaction with Tyr48 as well as improve hydrolytic stability and solubility of the compound (Figure 15). Unfortunately, the high affinity *N*-linked glycans were observed to anomerize from the active α-mannoside to the inactive β-mannoside in the acidic environment of the stomach [117,118]. To circumvent this anomerization, the amino group was replaced by various linkages (-OCH_2_-, -SCH_2_-, -CH_2_S-, -CH_2_CH_2_-, -OCH_2_CH_2_-, -CH_2_NH-), which had been slightly extended compared to the first generation in order to improve π-π stacking with Tyr48; this strategy afforded optimized substrate **49** [117,119].

Fluorination is well established to be an effective method for modulating the p*K*_a_ and p*K*_b_ properties of neighboring functional groups, thereby influencing the net charge of a molecule at physiological pH, the strength of ionic interactions, etc. In addition, it has been shown to enhance lipophilicity, and is often used in medicinal chemistry to block sites of undesired metabolism [100,120]. Although not widely used, potentially owing to its synthetic challenges, the fluorination of pyranose structures has been used to destabilize the oxocarbenium intermediate required for glycoside hydrolysis, thereby reducing the rate of metabolic degradation of glycomimetics.

### 3.2. Improving Oral Bioavailability

Native carbohydrates are not orally available due to their high hydrophilicity which prevents passive permeation across the intestinal membrane, but this can be improved by modifying carbohydrates to reduce their polar surface area. This can be achieved by using some of the techniques already discussed, such as deoxygenation of hydroxy groups or the addition of hydrophobic substituents. Appending a moiety which is known to undergo active transport into the bloodstream can also facilitate improved oral bioavailability. For example, specific amino acid sequences known to target peptide transporters (e.g., PEPT1, PEPT2) have been used; valacyclovir (Valtrex^®^), an antiviral drug targeting herpes simplex, contains the active component acyclovir (Zovirax^®^) conjugated to a valine which affords a five-fold increase in oral bioavailability [121,122].

Interestingly, pre-organization can also improve oral bioavailability, as it reduces the polar surface area and thereby substrates can more effectively permeate the membrane. This concept was nicely demonstrated in a study of peptides, where rigidified cyclic peptides were confirmed to have less exposed polar surface area when compared to more flexible cyclic peptides [123]. The rigidified peptides, with their buried polar groups, had better membrane permeability and metabolic stability.

**Figure 16:**
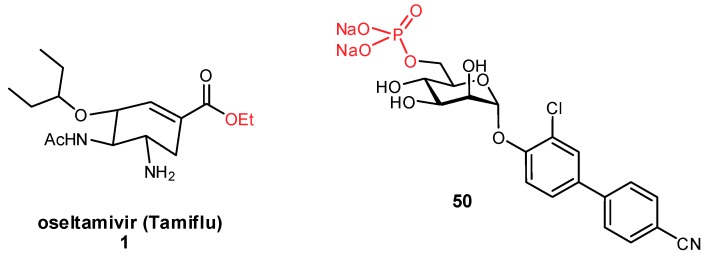
Prodrugs can be used to improve oral bioavailability, by improving factors such as intestinal epithelial membrane permeability (**1**, ester prodrug) or solubility (**50**, phosphate prodrug) [125,126].

An alternative to improving oral bioavailability is to use the prodrug approach [124]. By modifying a polar substrate with a hydrophobic moiety (e.g., through ester formation), the hydrophilicity becomes temporarily reduced enabling passive permeation through the membrane. Upon entry into the bloodstream, ubiquitous endogenous esterases can cleave the pro-moiety, unmasking the active component. With the condition that the esters are not cleaved prematurely by gastrointestinal esterases, this approach has been very important for improving the absorption of glycomimetic compounds, such as oseltamivir (Tamiflu^®^; Figure 16) [22,23,125]. Alternatively, for glycomimetics which have been modified with aromatic substituents to engage new protein interactions, they are often membrane permeable but suffer instead from poor aqueous solubility. In these instances, the prodrug approach can also be used to append a polar moiety which temporarily improves solubility. For example, phosphorylated prodrugs have been used in the development of FimH antagonists; this has proven a useful strategy for phosphorylated derivatives which undergo slower hydrolysis, as rapid hydrolysis can cause undesirable precipitation of the substrate (Figure 16) [24,126].

### 3.3. Improving Residence Times and Plasma Half-lives

**Figure 17:**
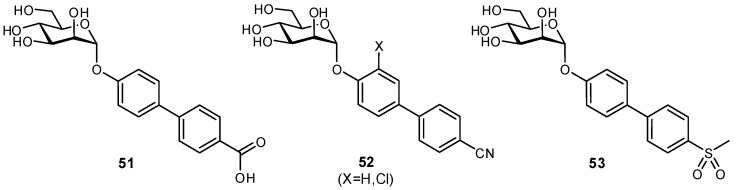
Bioisosteric replacement of a carboxylate moiety was used to optimize FimH antagonist **51** which suffered from rapid renal clearance and low tubular reabsorption in vivo [128].

The residence times of lectins with their physiological ligands is typically in the range of a few seconds. In order for carbohydrate-based molecules to be suitable as therapeutics, it is necessary to extend both the residence time and circulation times (from minutes to hours) [127]. Several strategies have been utilized to improve the plasma half-lives of glycomimetics and to reduce their rate of clearance from the bloodstream. For example, the FimH antagonist **51** displayed high affinity but poor therapeutic potential as it was rapidly eliminated by the kidneys and had low reabsorption by renal tubules [128]. These poor pharmacokinetic properties were attributed to the carboxylate moiety which had been introduced for enhancing π-π interactions between FimH and the biaryl aglycone. To improve therapeutic usefulness, bioisosteres of the carboxylate were generated to afford FimH antagonists with optimized pharmacokinetic profiles (Figure 17). In comparison to the original substrate, some bioisosteres with greater hydrophobicity (reduced desolvation penalty) and conformational rigidity (reduced entropic cost of binding) even displayed enhanced affinities.

Another approach involves removing functional groups from glycomimetics which enable active transport or are prone to metabolic degradation. For example, organic anion and cation transporters (OATs and OCTs, respectively) in the liver and kidneys can actively excrete certain glycoconjugates, and are often responsible for their very short half-lives [129]. OAT1 to OAT5 can recognize various anions connected to a hydrophobic ring. It has been demonstrated that the active oseltamivir metabolite is recognized by OAT transporters, and co-therapy with an OAT1 competitive inhibitor (probenecid) considerably improved serum half-life [130]. By elucidating which functional groups are responsible for active transport and/or metabolic degradation, these groups can be replaced by similar bioisosteres and/or modified by fluorination, an approach commonly used to block sites of cytochrome oxidation and other metabolic processes.

An alternative approach to increasing serum half-life is to append a moiety which is known to bind serum proteins, thereby increasing circulation time in the bloodstream. Plasma half-lives can be significantly extended when the carbohydrate ligand interacts with blood plasma components; for example, the heparins naturally bind to plasma proteins and display improved half-lives which make them more suitable for therapeutic use as anti-coagulants [131,132].

A somewhat drastic approach to glycomimetics is to completely replace the carbohydrate with a non-carbohydrate-based scaffold, and then to build in the essential functional groups while retaining their same spatial orientation as compared to the binding mode of the native ligand. Additional hydrophobic or charged moieties can also be appended to the scaffold to facilitate secondary interactions. A number of different scaffolds have been utilized in this approach to mimic either pyranoses or their enzymatic transition states, ranging from peptides to four-membered rings (such as oxetanes, azetidines, thietanes, and cyclobutanes; recently reviewed by Hazelard and Compain) [13,133,134,135,136,137,138]. Although potentially more difficult to design rationally, these scaffolds can offer additional advantages in terms of stability and more constrained structures (reducing entropic binding penalties). To identify novel scaffolds, the screening of molecule libraries has been utilized to identify potential new substrates which mimic the original glycan; these often rely on the generation of an antibody against the native glycan, with the antibody then used for screening to identify lead structures which can be further evaluated as competitive inhibitors. Although arguably less elegant, this strategy has been successful in identifying a mimic of Le^x^ which could elicit improvements in neuronal survival and neurite outgrowth [139], or mimetics of heparins which have been used to protect endothelial colony forming progenitor cells in diabetic patients [140], potentially leading to a solution for improved vascular endothelial repair and wound healing for foot ulcers. This approach has also been used in a glycomimetic-containing carbohydrate-based vaccine, where cross-reactive antibodies identified potential peptides through library screening, which were then more immunogenic, cross-reactive with the native substrate, and more easily obtainable through synthesis [141,142,143].

### 3.4. Other Considerations

Although methods to improve pharmacokinetic parameters have just been discussed, some of the approaches described may not be desirable for particular therapeutic targets. For example, in some glycomimetics, oral bioavailability is not required: the α-glucosidase inhibitors used to treat diabetes target proteins in the brush border of the small intestine, and therefore do not need to be passively transported across the intestinal membrane. This is also the case for neuraminidase inhibitors such as zanamivir (Relenza^®^) which can be used to target viral infections in the pharyngeal mucosa.

Similarly, for some glycomimetic therapeutics it could be desirable to have shorter serum half-lives; for example, the FimH antagonists discussed previously rely on renal excretion to reach their desired protein targets in the urinary tract. Although a faster renal clearance is desirable in the treatment of urinary tract infections, glycomimetics that are cleared too quickly would require too frequent dosing; therefore, some hydrophobicity is still desirable to facilitate renal tubular reabsorption and somewhat prolong circulation times.

## 4. Conclusions

Although historically carbohydrate-based therapeutics have not been overly successful in the drug development pipeline, there have been numerous successful approaches recently developed which can be used to circumvent the weak affinities and poor pharmacokinetic properties typically attributed to glycans. Given their extensive structural diversity and involvement in a broad range of diseases, carbohydrates and their associated glycomimetics have recently come back into focus as a very promising therapeutic option. By identifying an appropriate target and applying several of the strategies discussed in the context of this review, high affinity glycomimetics with favorable pharmacokinetic profiles can be developed, and eventually carried forward into the clinic.

## Figures and Tables

**Table 1:** Examples of bioisosteric groups.

Original Group	Potential Replacements